# Material Balance Probing of Aqueous Iron–Nickel Sulfate Solid–Liquid Equilibria from 279.2 to 283.2 K

**DOI:** 10.1021/acs.jced.5c00736

**Published:** 2026-07-03

**Authors:** Eduarda Depiné Dornelles Da Silva, Kentaro Oishi, Jean-Philippe Harvey, Julian Self

**Affiliations:** † Department of Chemical Engineering, 5596 Polytechnique Montreal, Montreal, Quebec H3C 3A7, Canada; ‡ Centre for Research in Computational Thermochemistry, Montreal, Quebec H3C 3A7, Canada

## Abstract

Solid–liquid equilibria of aqueous transition-metal sulfate solutions are relevant to hydrometallurgical processes, including those arising in the recycling of lithium-ion batteries. However, for many ternary systems, the solubility limits are known only in limited temperature ranges, and the nature of the solids formed is unresolved. We perform material balance solubility measurements combined with theoretical modeling to study the solubility limits of the FeSO_4_–NiSO_4_–H_2_O system, which involves solid solutions. We characterize the composition of the solid solutions from 279.2 to 283.2 K using chemical analysis and powder X-ray diffraction. The agreement between the thermodynamic model and experimental compositions for the liquid and solid phases shows that the proposed equilibrium constants, ideal solid solution model, and Pitzer equations employed allow fair modeling of phase equilibria.

## Graphical abstract

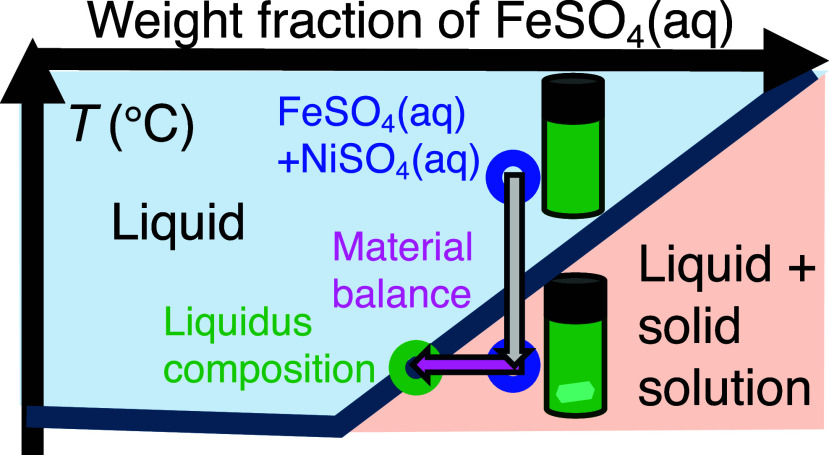

## Introduction

The motivation for our study of sulfate solutions is the recycling of lithium-ion battery batteries, where one of the main recycling processes is the sulfate hydrometallurgical route. In this process, liquid sulfate aqueous solutions are created as a result of the leaching step with the goal of obtaining selected metal solid sulfate salt hydrates MSO_4_·*n*H_2_O.^1^ Here, M is a metal of interest, such as Ni, Co, Mn, Fe, or others which can exist in solution as an ion M^*z*+^. In many cases, due to a second metal ion N^*z*+^ in solution, it is difficult to obtain pure salts via chemical precipitation. Such difficulties are compounded by the fact that, for many of the sulfate systems, thermodynamic phase equilibria data are unknown or unreported, for example, solubilities as a function of temperature at ambient pressure. In fact, high-precision solid–liquid thermodynamic data are of fundamental importance in designing chemical precipitation unit operations to efficiently separate valuable metallic ions from end-of-life Li-ion battery black mass.^2^ In this work, we investigate the solubilities of a ternary system of M = Fe and N = Ni ions in solution, noting that practical leachate solutions are often complex with three or more different cations present. We chose this system as an initial case study in the effort to collect and evaluate previously unavailable solubility data. As far as we know, no phase diagram which includes both solubility limits and tie lines has been reported for this system, motivating our investigation. To allow thermodynamic modeling of tie lines, we report revised solubility constants for iron sulfate in morenosite and nickel sulfate in melanterite. Finally, we note that we experimentally probe solubility limits in a previously unstudied temperature range, from 279.2 to 283.2 K.

Ternary transition-metal sulfate systems have been the subject of both recent and past studies. Many research efforts have focused on metal-acid ternary systems.^3–5^ In such systems, generally, the solid–liquid equilibria are determined by solids of constant composition (also called stoichiometric compounds). In other systems, for example, ternary aqueous sulfates with two different transition metal ions, the solid–liquid equilibria are often determined by solid solutions in which transition metal ions can substitute, at least partially, on given crystallographic sites.^6–9^ However, the aqueous solubility of sulfate solid solutions which hold multiple transition metal ions has been the subject of fairly limited scholarship.^7,10^ In the case of NiSO_4_ and FeSO_4_, as far as we know, only Charykova et al. (2010)^10^ have published solubility data and solubility constants of the solid solution. Nonetheless, the biphasic solid compositional region for the solid solution has been previously reported.^8,11,12^ As is consistent with previous literature, we will refer to the biphasic solid region as a miscibility gap, noting that the two solid phases present in the miscibility gap are composed of different crystal structures.

## Solid–Liquid Equilibria

At sufficiently low metal sulfate concentrations, liquid electrolyte/ice equilibrium determines the liquidus via the following equilibrium relation:

1 $$H_{2}O\left(s\right)⇌H_{2}O\left(l\right)$$

At sufficiently high ionic concentrations, the liquidus is determined by the solubility of hydrates. While studying a temperature window from 279.2 to 283.2 K, we assume in this work that the solubility constants of relevance are heptahydrates,^10^ such that the two relevant solids are Fe_*x*_Ni_1–*x*_SO_4_·7H_2_O (s, melanterite basis) and Fe_*x*_Ni_1–*x*_SO_4_·7H_2_O (s, morenosite basis). These are solid solutions of iron and nickel sulfates with two different crystal structures, that of the melanterite monoclinic structure and that of the morenosite orthorhombic structure. Iron, as part of a melanterite basis, can be described by the following solubility equilibrium:

2 $${\mathrm{FeSO}}_{4}\cdot 7H_{2}O\left(s,\mathrm{melanterite}\;\mathrm{basis}\right)⇌Fe^{2+}\left(aq\right)+{{\mathrm{SO}}_{4}}^{2-}\left(aq\right)+7H_{2}O\left(l\right)$$

The solubility of nickel sulfate in the solid solution can also be expressed via a similar equilibrium:

3 $${\mathrm{NiSO}}_{4}\cdot 7H_{2}O\left(s,\mathrm{melanterite}\;\mathrm{basis}\right)⇌{\mathrm{Ni}}^{2+}\left(\mathrm{aq}\right)+{{\mathrm{SO}}_{4}}^{2-}\left(\mathrm{aq}\right)+7H_{2}O\left(l\right)$$

At sufficiently high nickel sulfate concentrations, the solid solution may hold a morenosite basis. The solid–liquid equilibrium with aqueous iron ions and the solid solution can be expressed as

4 $${\mathrm{FeSO}}_{4}\cdot 7H_{2}O\left(s,\mathrm{morenosite}\;\mathrm{basis}\right)⇌{\mathrm{Fe}}^{2+}\left(\mathrm{aq}\right)+{{\mathrm{SO}}_{4}}^{2-}\left(\mathrm{aq}\right)+7H_{2}O\left(l\right)$$

Finally, the solid–liquid equilibrium with aqueous nickel ions and this solid solution can be expressed as

5 $${\mathrm{NiSO}}_{4}\cdot 7H_{2}O\left(s,\mathrm{morenosite}\;\mathrm{basis}\right)⇌{\mathrm{Ni}}^{2+}\left(\mathrm{aq}\right)+{{\mathrm{SO}}_{4}}^{2-}\left(\mathrm{aq}\right)+7H_{2}O\left(l\right)$$

For equilibria [Disp-formula eq2] and 5, the solubility constants have been reported by various groups.^10,13,14^ For nickel in a melanterite basis or iron in a morenosite basis, solubilities at 293.2 and 298.15 K have been modeled, and solubilities at 293.2 K have been reported.^10^ However, we were unable to obtain the source publications of the measurements reported in Charykova et al. (2010) due to current library limitations. We also note that they do not directly report the composition of the solid solutions as a function of liquid electrolyte composition. At higher temperatures (*T* > 303.2 K), it is likely that the solid–liquid equilibria involve other hydrates, as they are part of the known phase diagrams for the corresponding binary systems.^13–15^

It is our goal to experimentally collect solid-solution aqueous-solution equilibria data and evaluate it against equilibria data computed from the literature-derived equilibrium constants. Using a methodology relying on material balance, we carried out solubility measurements from 279.2 to 283.2 K, which we compared to solubility data generated from a thermodynamic model.

## Experimental Procedure

### Experimental Materials

In this study, two different sulfates were used as received (AR) to obtain the solutions: iron sulfate heptahydrate FeSO_4_·7H_2_O, 99.5% pure, purchased from Thermo Scientific, and nickel sulfate heptahydrate NiSO_4_·7H_2_O, 99% pure, commercialized by Sigma-Aldrich. Table 1 provides further details regarding the reagents used. The solvent used was deionized water, which was obtained using a Milli-Q EQ 700 water purifier.

**Table 1:** Details of the Sulfates Used in This Study

Chemical name	CAS	Molecular weight	Specification	Analytical method	Supplier
Iron(II) sulfate heptahydrate	7782-63-0	278.01	AR, ≥99.5%	Titration with KMnO_4_	Thermo Scientific
Nickel(II) sulfate heptahydrate	10101-98-1	280.86	AR, ≥99.0%	Titration with EDTA	Sigma-Aldrich

### Solution Preparation

The liquid electrolyte solutions were prepared in a glass cell (Figure 1) inside of a fume hood. The pressure was atmospheric, which is assumed to be the relevant pressure for the present work. The temperature was maintained at 303.2 K and controlled by a circulating water bath (III) (Lindbergh Blue M, Thermo Scientific) and monitored by a thermocouple  (IV) (EasyView 11A, Extech). In addition, the cell was connected to a nitrogen bubbler (I) (300 mL/min) and to a magnetic stirrer (350 rpm). Figure 1 shows how the cell prevents solution contact with oxygen via a pressure difference. All openings were properly closed except for opening II, where there is a tube responsible for transporting excess gases from inside the cell to an aqueous environment. This ensures that no other gases (besides those supplied by the bubbler) can enter and that the pressure will remain constant during the preparation of the solutions.

**Figure 1:**
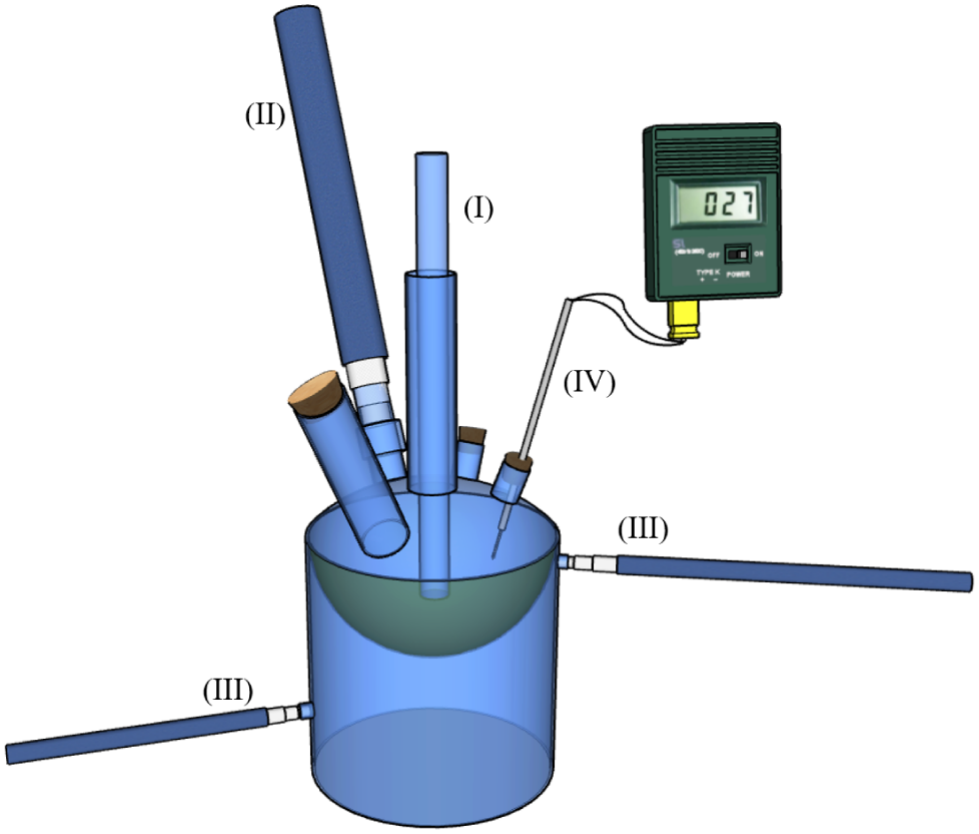
Schematic design of the setup used to prepare the solutions. I) Refers to the nitrogen bubbler; II) tube for excess gases transportation; III) circulating water bath; and IV) thermocouple.

First, a known concentration of solvent was weighed in a balance (Entris II Sartorius, with an accuracy of 0.0001 g) and poured into the cell. To ensure a negligible presence of oxygen in addition to temperature homogeneity, the solvent was bubbled while being stirred for 40 min. This was undertaken to prevent^16^ Fe­(II) oxidation to Fe­(III) due to the presence of oxygen in the aqueous solution. The latter can precipitate as a hydroxide, changing the color of the solution to brown.^17–19^ Previous studies have focused on characterizing the kinetics of this reaction under different conditions, mainly in terms of pH and temperature differences. It was found that the lower the pH and temperature of the solution, the slower the oxidation reaction.^17–19^ The solutes were then weighed and introduced into the cells. Stirring and N_2_ bubbling were stopped for 5 min to allow the addition of the solutes, and stirring and bubbling resumed afterward. After 2 h, the water bath circulation was stopped, and the solution was transferred to a nitrogen-filled glove bag (Sigma-Aldrich) so that the solution could be handled with minimal oxygen concentration (<2 v %) in the vials where liquid–solid equilibria will be inspected and studied. The time required to completely dissolve the solute was determined by preliminary experiments using an aqueous solution containing 20 wt % iron sulfate, which consisted of measuring the chemical composition via AAS (atomic absorption spectroscopy) at intervals of 1 h 30 min, 2 h, 3 h, and 17 h.

### Solubility Measurement

The measurement of solubilities of sulfate solutions is, in practice, somewhat challenging. We first considered using DSC (differential scanning calorimetry) to probe liquidus temperatures. Unfortunately, we were unable to reliably obtain crystallization of the expected solid, even with the addition of nanoinoculants to promote precipitation. Following the hypothesis that this is due to the small sample size of the pans (7.5 μL of sample), which prevented the formation of precipitation nuclei of critical size (assuming that nucleation is a stochastic process^20^), we increased the container size by a factor of more than 650 by using 5 mL vials. This allowed the formation of crystals in the vials once the temperatures below the liquidus were held for sufficiently long. It is expected that, in the concentration ranges studied, the solid–liquid equilibria are determined by transition metal hydrates, which was confirmed via crystallographic analysis. Crystalline structure was analyzed using X-ray diffraction (XRD) (Rigaku, MiniFlex600). In this case, it was necessary to crush the crystals into fine powder using a porcelain mortar and pestle, and then place them in a zero-background sample holder. XRF (fluorescence X-ray) reduction energy mode was employed with an angular range of 3° to 120°, with a step of 0.01° and speed 0.1°/min. The diffraction patterns were analyzed using Le Bail refinement through the Profex software. The spectra available in the Crystallography Open Database (COD) were used as references, specifically: 9004773 (melanterite), 9011883 (morenosite), 9007473 (rozenite), and 9011367 (nickelhexahydrite).^21^ Given the difficulty of deconvoluting the XRD signal which holds overlapping contributions from potentially various hydrates, we used the simpler Le Bail refinement over conventional Rietveld refinement.^22^ This allowed identification of the crystal structures present, but not of the exact quantities present, a task which is outside the scope of the current work. The crystals were placed on glass Petri dishes and subjected to morphological analysis using a VHX-7000 optical microscope (Keyence) with lenses ranging from ×20 to ×80 magnification.

We first prepared the solutions above the liquidus temperature (303.2 K) and verified visually that all the solution was liquid. To ensure the homogeneity of the solution, the 5 mL of solution was handled using a syringe with a 0.22 μm filter at its tip. Afterward, we placed the samples in a temperature-controlled chamber at a temperature below the expected liquidus for 1 day to a week. The waiting time needs to be sufficiently long to allow reaching thermodynamic equilibrium, while recognizing that much longer waiting times could lead to oxygen contamination risks. We note that previous work on this chemical system successfully utilized waiting times of 20 h to reach thermodynamic equilibrium after cooling,^12^ which is similar to the waiting time used in this work. Solutions were visually verified to not adopt a brown color which would be indicative of Fe­(III) formation.

Following the formation of one or many crystals, the vial was weighed before and after the vacuum filtration of the crystals, followed by weighing of the crystals only. Preliminary experiments were conducted to analyze the ratio between the concentration of iron sulfate and nickel sulfate present in the crystals after 1 day, 2 days, 1 week, and 2 weeks of formation for four different ternary compositions. Longer times did not show a significant change in the solid iron-to-nickel ratio (less than 30%). A duplicate solution was prepared of the solution above the liquidus temperature containing 5 wt % of iron sulfate and 25 wt % of nickel sulfate, achieving solubility limits similar to those measured in the first round (within 0.13 wt % for FeSO_4_ and 0.8 wt % for NiSO_4_). Provided we have reached thermodynamic equilibrium, and the chemical composition of the crystal is known or estimated, material balance allows for the calculation of the liquidus composition. Accordingly, the measurement of liquidus composition at a given temperature is possible. We describe this general methodology as material balance, noting that it is one of many possible solubility measurement methods.^23^ The details of the material balance calculations are in the following subsections. We chose to study solid–liquid equilibria from 279.2 to 283.2 K, as this allowed the practical study of systems of moderate sulfate concentrations (≤25 wt %) through material balance probing, which requires a liquidus temperature sufficiently above the studied temperature for a given concentration.

### Material Balance

#### Binary Material Balance

Figure 2 illustrates the crystallization process used in our measurement of solubility for a binary sulfate electrolyte. Specifically, a liquid aqueous electrolyte, once cooled below the liquidus, will separate into a solid crystal phase and a liquid phase of composition equal to that of the liquidus at that temperature. This process, using material balance, allows us to measure the liquidus composition. What is initially known are the masses of H_2_O and FeSO_4_ in the liquid solution at temperatures higher than the liquidus: $w_{H_{2}O}$ and $w_{{\mathrm{FeSO}}_{4}}$ . We note that these are also effectively the system compositions. Once we cool below the liquidus, a crystal is formed with measurable mass $w_{{\mathrm{FeSO}}_{4}\cdot 7H_{2}O}^{\mathrm{crystal}}$ . This mass can be resolved into the masses of H_2_O and FeSO_4_ in the crystal $w_{H_{2}O}^{\mathrm{crystal}}$ and $w_{{\mathrm{FeSO}}_{4}}^{\mathrm{crystal}}$ using the assumed stoichiometry of FeSO_4_·7H_2_O:

6 $$w_{{\mathrm{FeSO}}_{4}\cdot 7H_{2}O}^{\mathrm{crystal}}=n_{{\mathrm{FeSO}}_{4}}^{\mathrm{crystal}}M_{{\mathrm{FeSO}}_{4}}+n_{H_{2}O}^{\mathrm{crystal}}M_{H_{2}O}$$

7 $$7n_{{\mathrm{FeSO}}_{4}}^{\mathrm{crystal}}=n_{H_{2}O}^{\mathrm{crystal}}$$

In the above equations, *n*_*i*_ refers to the number of moles of species *i*, which allows calculation of the mass of the species through *n*_*i*_ *M*_*i*_ = *w*_*i*_, where *M*_*i*_ is the molar mass. The weights of the salt and the water in the liquid solution at the liquidus composition, denoted $w_{{\mathrm{FeSO}}_{4}}^{\mathrm{liquidus}}$ and $w_{H_{2}O}^{\mathrm{liquidus}}$ , can be resolved via the two following material balance equations:

8 $$w_{H_{2}O}\left(T>T_{\mathrm{liquidus}}\right)=w_{H_{2}O}^{\mathrm{liquidus}}+w_{H_{2}O}^{\mathrm{crystal}}$$

9 $$w_{{\mathrm{FeSO}}_{4}}\left(T>T_{\mathrm{liquidus}}\right)=w_{{\mathrm{FeSO}}_{4}}^{\mathrm{liquidus}}+w_{{\mathrm{FeSO}}_{4}}^{\mathrm{crystal}}$$

**Figure 2:**
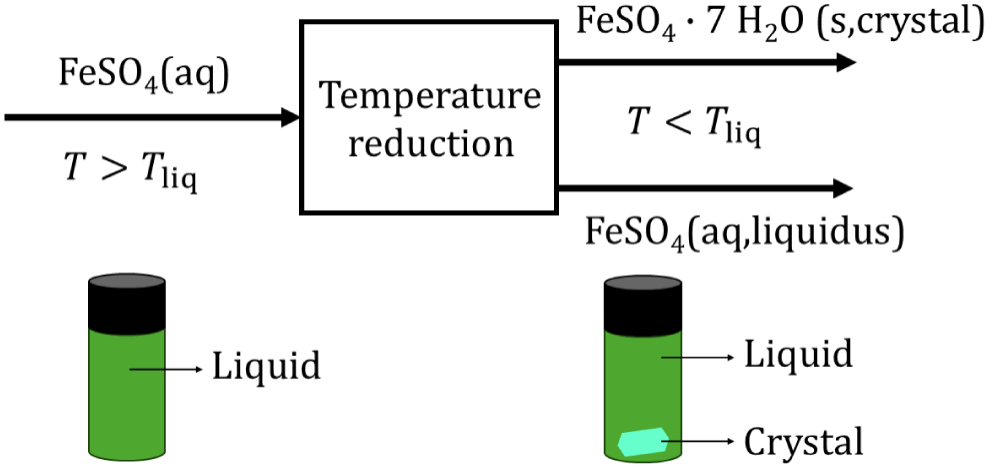
Crystallization schematic for a binary sulfate electrolyte.

#### Ternary Material Balance

The treatment for the binary solution can be generalized for electrolyte systems which are in equilibrium with a nickel–iron sulfate solid solution. Figure 3 illustrates crystallization for the ternary iron sulfate nickel sulfate water system. In this case, we assume a solid solution is formed in a phase distinct from the liquid aqueous phase for which nickel sulfate and iron sulfate are at the relevant liquidus composition. Equations 6 and 7 can be generalized to the three following equations, since the solid solution holds both sulfates:

10 $$w_{{\mathrm{Fe}}_{x}{\mathrm{Ni}}_{1-x}{\mathrm{SO}}_{4}\cdot 7H_{2}O}^{\mathrm{crystal}}=n_{{\mathrm{FeSO}}_{4}}^{\mathrm{crystal}}M_{{\mathrm{FeSO}}_{4}}+n_{{\mathrm{NiSO}}_{4}}^{\mathrm{crystal}}M_{{\mathrm{NiSO}}_{4}}+n_{H_{2}O}^{\mathrm{crystal}}M_{H_{2}O}$$

11 $$7\left(n_{{\mathrm{FeSO}}_{4}}^{\mathrm{crystal}}+n_{{\mathrm{NiSO}}_{4}}^{\mathrm{crystal}}\right)=n_{H_{2}O}^{\mathrm{crystal}}$$

12 $$\left(1-x\right)n_{{\mathrm{FeSO}}_{4}}^{\mathrm{crystal}}=xn_{{\mathrm{NiSO}}_{4}}^{\mathrm{crystal}}$$

*x*/(1 – *x*) is the mole ratio of iron sulfate to nickel sulfate, which can be experimentally resolved if the mass ratio of iron sulfate to nickel sulfate can be measured. In addition to eqs 8 and 9, there is a material balance equation for nickel sulfate, which allows resolution of the nickel sulfate content at the liquidus composition, provided we know the initial nickel sulfate content of the solution above the liquidus temperature and the nickel content in the crystal:

13 $$w_{{\mathrm{NiSO}}_{4}}\left(T>T_{\mathrm{liquidus}}\right)=w_{{\mathrm{NiSO}}_{4}}^{\mathrm{liquidus}}+w_{{\mathrm{NiSO}}_{4}}^{\mathrm{crystal}}$$

Equations 8 to 13 allow the resolution of the composition of the liquid in equilibrium with the solid solution, provided the masses of the crystal and liquid below the liquidus temperature are measured and the ratio of iron sulfate to nickel sulfate in the solid solution is known. This ratio can be either calculated from a thermochemical model of the solid solution or measured via a chemical analysis.

**Figure 3:**
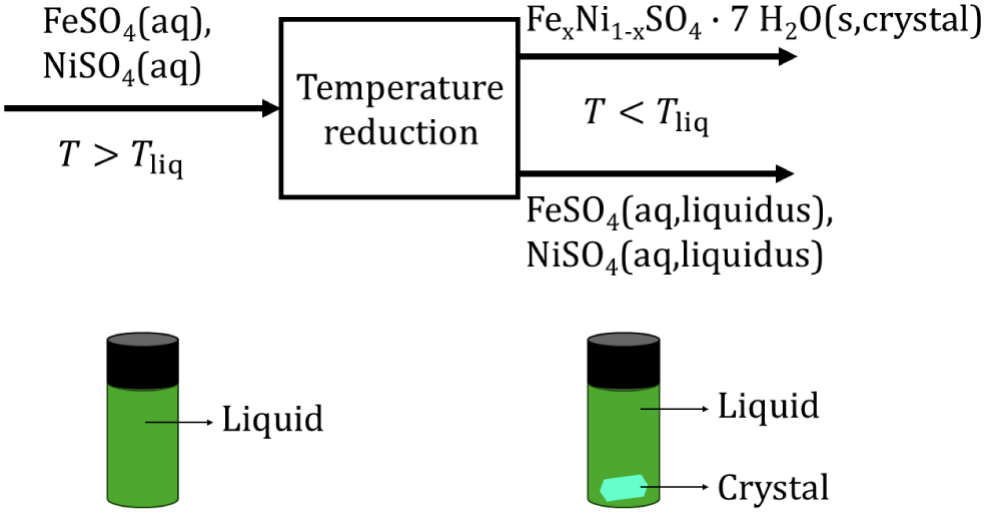
Crystallization schematic for a ternary sulfate electrolyte.

### Chemical Analysis (Atomic Absorption Spectroscopy)

The chemical composition analysis was performed using AAS with a PerkinElmer PinAAcle 900F spectrometer. To ensure that all the analyses were within the equipment’s measurement range, all the samples were diluted by a volume factor of 10,000 using an aqueous solution of 3 v% nitric acid (HNO_3_). Two successive dilution steps were used to ensure better dilution accuracy.

Once the crystal formed, the entire crystal present in the vial was dissolved at room temperature in 5 mL of deionized water. To ensure homogeneity of the aliquot analyzed, 1 mL of solution was separated using a syringe with a 0.22 μm pore filter. An aliquot of 120 μL was taken from the filtered liquid solution and diluted in 12 mL of HNO_3_. A new aliquot of 120 μL was then taken from this new solution and diluted again as previously described.

## Model Thermodynamics

### Solid Solution Miscibility Gap

Table 4 shows the solubility constants in logarithmic form for Fe in melanterite and Ni in morenosite, calculated from thermodynamic data provided in Kobylin et al. (2011)^13^ and Kobylin (2011).^14^ In order to model the relevant solid solutions, we followed Charykova et al.’s (2010) assumption of an ideal Ni and Fe solution in the melanterite (FeSO_4_·7H_2_O) or morenosite (NiSO_4_·7H_2_O) host lattices for simplicity.^10^ However, we did not use their solid solution solubility constants (see Table 4) as these did not adequately reproduce the miscibility gap of the solid solutions.^8^ The miscibility gap of the morenosite and melanterite structures has been reported as occurring above 0.19 mole fraction Fe content versus Ni in morenosite (i.e., below 0.81 Ni) and below 0.54 Fe content in melanterite (above 0.46 Ni).^8,12^ These values, reported by Balarew et al.,^12^ are consistent with those later reported by Siebke et al.^24^ In the miscibility gap, liquid + melanterite + morenosite equilibrium involves two solids which are at the fixed composition determined by the miscibility gap and which are at equilibrium. In the case where the energetics in the transition metal solid solutions are approximated as ideal, the solubility constants of the equilibria 2, 3, 4, and 5 determine the miscibility gap. Conversely, provided the binary solution solubility constants are known (relevant to equilibria 2 and 5), the two other solubility constants can be determined if the miscibility gap is known. Since we assume that a thermodynamic equilibrium is established between both solid solutions (i.e., melanterite and morenosite), the following chemical potential (μ_species_^phase^) equality for the FeSO_4_·7H_2_O system component holds:

14 $$\mu_{{\mathrm{FeSO}}_{4}\cdot 7H_{2}O}^{\mathrm{melanterite}}{|}_{\mathrm{misc.}\;\mathrm{gap}}=\mu_{{\mathrm{FeSO}}_{4}\cdot 7H_{2}O}^{\mathrm{morenosite}}{|}_{\mathrm{misc.}\;\mathrm{gap}}$$

The same equilibrium condition also holds for the NiSO_4_·7H_2_O system component, specifically:

15 $$\mu_{{\mathrm{NiSO}}_{4}\cdot 7H_{2}O}^{\mathrm{morenosite}}{|}_{\mathrm{misc.}\;\mathrm{gap}}=\mu_{{\mathrm{NiSO}}_{4}\cdot 7H_{2}O}^{\mathrm{melanterite}}{|}_{\mathrm{misc.}\;\mathrm{gap}}$$

Expanding the above equations, we can write the two following equations relating the composition of the structures in the miscibility gap to the various solubility constants, *K*_SP_, where mel is short for melanterite and mor for morenosite:

16 $$\ln K_{\mathrm{SP}}\left(\mathrm{Fe},\mathrm{mel}\right)+\ln\left(\frac{n_{\mathrm{Fe}}^{\mathrm{mel}}}{n_{\mathrm{Fe}}^{\mathrm{mel}}+n_{\mathrm{Ni}}^{\mathrm{mel}}}\right){|}_{\mathrm{misc}.\mathrm{gap}}=\ln K_{\mathrm{SP}}\left(\mathrm{Fe},\mathrm{mor}\right)+\ln\left(\frac{n_{\mathrm{Fe}}^{\mathrm{mor}}}{n_{\mathrm{Fe}}^{\mathrm{mor}}+n_{\mathrm{Ni}}^{\mathrm{mor}}}\right){|}_{\mathrm{misc}.\mathrm{gap}}$$

17 $$\ln K_{\mathrm{SP}}\left(\mathrm{Ni},\mathrm{mel}\right)+\ln\left(\frac{n_{\mathrm{Ni}}^{\mathrm{mel}}}{n_{\mathrm{Fe}}^{\mathrm{mel}}+n_{\mathrm{Ni}}^{\mathrm{mel}}}\right){|}_{\mathrm{misc}.\mathrm{gap}}=\ln K_{\mathrm{SP}}\left(\mathrm{Ni},\mathrm{mor}\right)+\ln\left(\frac{n_{\mathrm{Ni}}^{\mathrm{mor}}}{n_{\mathrm{Fe}}^{\mathrm{mor}}+n_{\mathrm{Ni}}^{\mathrm{mor}}}\right){|}_{\mathrm{misc}.\mathrm{gap}}$$

Following the reported miscibility gap,^8^ we solved for ln *K*_SP_(Fe,mor) and ln *K*_SP_(Ni,mel) values of −4.2155 and −4.4542, as shown in Table 4. These values differ from Charykova et al. (2010), which, although they allow for reasonable modeling of solubility limits, will not reasonably reproduce the miscibility gap. A solid solution has three main entropic contributions: configurational, vibrational, and electronic. The first is accounted for in this work for Ni–Fe solid solutions through an ideal solution model, as in eqs 16 and 17. Vibrational entropic contributions are considered relatively composition-independent,^25^ while electronic contributions are likely negligible for the nonconductive metal sulfate solid solutions. Consistent with this rationale, we assumed that the terminal compositions of a given solid solution held the same *S* ^0^(298 K) and heat capacity, while the entropic effects of the intermediate compositions were accounted for exclusively by an ideal solution model with one sublattice. Tables 5 and 6 show the various employed entropies and heat capacities, respectively. This allowed for the calculation of an enthalpy of formation for Ni in melanterite and Fe in morenosite, as shown in Table 5, from the solid solution solubility constants used in the present work (see Table 4).

### Liquid Electrolyte Modeling

The binary NiSO_4_(aq) and FeSO_4_(aq) electrolytes and their solubilities were modeled following Kobylin et al. (2011) and Kobylin (2011).^13,14^ We used their reported enthalpies of formation, entropies, and heat capacities for solid compounds and ions in solution, as shown in Tables 5 and 6. For the entropies and formation enthalpies of heptahydrated iron sulfate in morenosite and heptahydrated nickel sulfate in melanterite, we used the values in Table 5, which were estimated as discussed in the previous section. The water heat capacity parameters were taken from the FactSage database, as shown in Table 6.^26^ The nonidealities of the liquid aqueous electrolyte were described using the Pitzer equations. The thermodynamic parameters are shown in Tables 6, 7, and 8. In order to model the solubility in the ternary system, we used liquid electrolyte ternary interaction parameters from Charykova et al. (2010), which are shown in Table 9. We chose these as they were the only available literature ternary parameters for this system, and they allowed fair reproduction of solubility limits. The present work’s model was implemented in FactSage, which allowed calculation of the ternary system’s phase equilibria.^26^

## Results

Figure 4 shows the liquidus line for system compositions of 5.00 wt % nickel sulfate computed with the model solid solution (blue) and with the assumption of nickel-free melanterite (dashed line). A solution was prepared at 17.0 wt % FeSO_4_ and 5.00 wt % NiSO_4_ at 303.2 K, which is above the liquidus temperature (red upside-down triangle). We cooled the solution vial below the liquidus line to 281.2 K. After 24 h, crystals were present, indicating the presence of solid–liquid equilibrium (green square). We note that this is consistent with the solid solution liquidus line shown. Once a crystal is formed, the liquid electrolyte composition changes. Using the material balance of a crystal observed for a system of 17.0 wt % of FeSO_4_ in solid–liquid equilibrium, it was found that the liquid is at a composition of 14.3 wt % FeSO_4_ in the solution, a quantity resolved using eqs 8 to 13. Such a quantity compares particularly well to the model-computed liquidus composition value of 14.0 wt % at the temperature 281.2 K. For nickel sulfate, the initial composition is 5.00 wt % and the liquidus composition is 4.9 wt % (computed and measured via material balance; see Table 2). Generally, since this is a ternary system, the system composition of nickel sulfate will be different from that of the resulting liquidus and requires special resolution, for example, through the use of tie lines. We also note that in the present work’s methodology, first the solid composition is experimentally determined (e.g., through AAS), which then allows material balance resolution of the liquidus composition.

**Figure 4:**
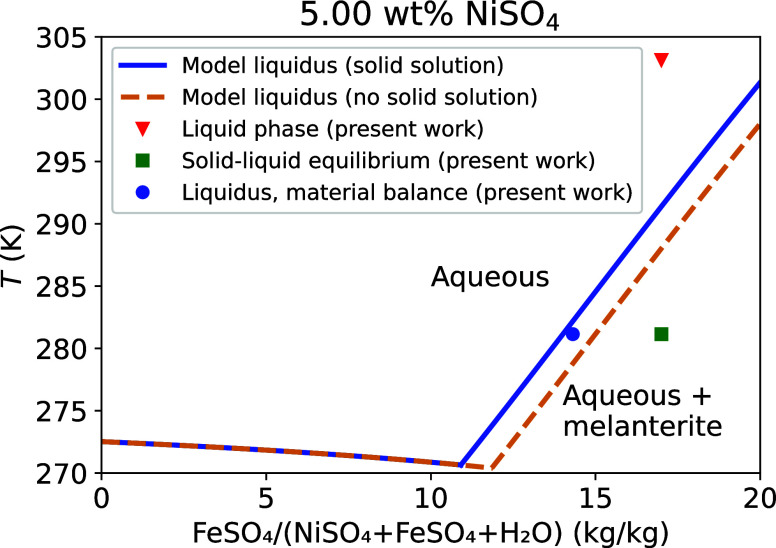
Liquidus line along a fixed nickel sulfate weight percent of 5.00% as a function of temperature. The pressure is atmospheric (100 ± 3 kPa). Numerical values are listed in Table 2.

**Table 2:** Liquidus Solution Composition ( $\frac{w_{{\mathrm{FeSO}}_{4}}^{\mathrm{liquidus}}}{w^{\mathrm{liquidus}}}$ , $\frac{w_{{\mathrm{NiSO}}_{4}}^{\mathrm{liquidus}}}{w^{\mathrm{liquidus}}}$ ) at Various Temperatures as a Function of Iron and Nickel Sulfate Composition of System ( $\frac{w_{{\mathrm{FeSO}}_{4}}}{w_{\mathrm{Tot}}}$ , $\frac{w_{{\mathrm{NiSO}}_{4}}}{w_{\mathrm{Tot}}}$ ) at Atmospheric Pressure (100 kPa)^a^

*T*	$\frac{w_{{\mathrm{FeSO}}_{4}}}{w_{\mathrm{Tot}}}$	$\frac{w_{{\mathrm{NiSO}}_{4}}}{w_{\mathrm{Tot}}}$	$\frac{w_{{\mathrm{FeSO}}_{4}}^{\mathrm{liquidus}}}{w^{\mathrm{liquidus}}}$		$\frac{w_{{\mathrm{NiSO}}_{4}}^{\mathrm{liquidus}}}{w^{\mathrm{liquidus}}}$	
				
(K)	(wt %)	(wt %)	(wt %)	(wt %)	(wt %)	(wt %)
			mat. bal.	model	mat. bal.	model
279.2	20.0	0	16.6	16.1	0	0
279.2	17.01	5.00	13.6	13.5	4.9	4.9
279.2	15.99	7.01	13.2	12.4	6.8	6.9
279.2	15.00	10.0	15.0	10.8	10.0	9.8
279.2	11.01	15.0	10.3	8.1	14.6	14.6
279.2	10.00	18.6	8.2	7.3	17.8	16.1
279.2	8.00	18.6	7.9	7.3	17.7	16.1
279.2	6.99	20.0	6.9	6.4	19.9	17.0
279.2	5.00	25.0	4.9	4.3	23.5	19.1
279.2	5.00	25.0	5.0	4.3	24.3	19.1
279.2	1.01	25.0	0.95	0.94	23.7	22.5
279.2	0	25.0	0	0	24.0	23.6
281.2	17.00	5.00	14.3	14.0	4.9	4.9
281.2	15.00	10.0	12.1	11.2	9.6	9.8
283.2	10.00	18.6	8.2	7.4	18.3	17.3
281.2	8.00	18.6	7.7	7.3	17.7	16.7

a Standard uncertainty: *u*(*T*) = 0.6 *K, u*(*P*) = 3 kPa. Relative uncertainties on measured quantities: $u_{r}\left(\frac{w_{{\mathrm{FeSO}}_{4}}}{w_{\mathrm{Tot}}}\right)=0.005$ ; $u_{r}\left(\frac{w_{{\mathrm{NiSO}}_{4}}}{w_{\mathrm{Tot}}}\right)$ = 0.01, $u_{r}\left(\frac{w_{{\mathrm{FeSO}}_{4}}^{\mathrm{liquidus}}}{w^{\mathrm{liquidus}}}\right)=0.04$ , $u_{r}\left(\frac{w_{{\mathrm{NiSO}}_{4}}^{\mathrm{liquidus}}}{w^{\mathrm{liquidus}}}\right)=0.04$ .

Figure 5 shows the sulfate salt solubilities as a function of total nickel sulfate and iron sulfate weight percentage at various temperatures. As for the previously detailed system, the solid–liquid equilibrium was characterized by material balance, following the presence of crystals in the liquid vial after a period of 1 to 7 days when its temperature was lowered below the liquidus temperature for each of the samples. For the binary systems (either 0 wt % FeSO_4_ or NiSO_4_), the data from Linke (1965) (squares)^15^ show good agreement with the model data, as expected. The measurements and subsequent material balance (circles), carried out with eqs 8 to 13, can be compared with the thermodynamic model data. Table 2 shows the numerical values of the calculated solubility compositions, denoted as weight percentages $\frac{w_{{\mathrm{FeSO}}_{4}}^{\mathrm{liquidus}}}{w^{\mathrm{liquidus}}}$ and $\frac{w_{{\mathrm{NiSO}}_{4}}^{\mathrm{liquidus}}}{w^{\mathrm{liquidus}}}$ . The average FeSO_4_ and NiSO_4_ disagreements for liquidus compositions are 0.8 and 1.3 wt %, respectively. This is comparable to the Charykova et al.^10^ data scatter, which is of 0.7 and 0.8 wt % in FeSO_4_ and NiSO_4_. For our data, the main sources of estimated experimental error are from the AAS measurement and the purity of the purchased salts. We also include a duplicate measurement of the system at 5.00 wt % FeSO_4_ and 25.0 wt % NiSO_4_ to demonstrate repeatability error. One data point, at 24.3 wt % NiSO_4_ and 4.9 wt % FeSO_4_, shows noticeable disagreement with the model solubility by 5 wt % in nickel sulfate. Although high, this is on the same order of magnitude as the highest errors from the Charykova data (2 wt %). Assuming the thermodynamic model has errors of similar magnitude to those from the experiment, we conclude that the collected data is generally in fair agreement with the model-predicted solubilities at low temperatures (279.2 to 283.2 K). Two sets of errors are present in the model, one originating from the ternary liquid solution Pitzer parameters (and equations) and the other from the ideal solution approximation. As no experimental activity data is as of yet available for either solution, the model error evaluation in this work is constrained to comparison with experimental solid–liquid equilibria, such as solubility, where both error sets are convoluted. Vapor pressure measurements of the saturated aqueous solution in further work could allow deconvolution of both error sets. The accurate thermodynamic modeling of solid solution-aqueous solution equilibria remains a challenging problem.

**Figure 5:**
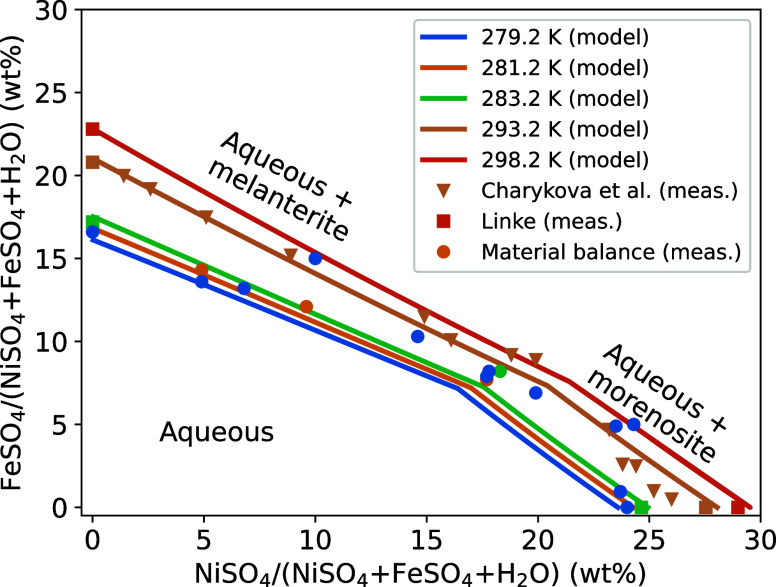
Solubilities as a function of total iron sulfate and nickel sulfate weight percentages in the mixture at various temperatures. The pressure is atmospheric (100 ± 3 kPa). Numerical values are listed in Table 2.

Figure 6 shows the boundary regions (black lines) of different phases present as a function of composition at 279.2 K. As samples were prepared in the liquid phase and cooled sufficiently to allow solid–liquid equilibrium, a solid phase appeared. The resulting compositions of liquid and solid phases were computed via the model (squares and diamonds), along with their respective tie lines (dashed lines). These compositions were also calculated through experimental material balance measurements (circles and upside-down triangles) with the corresponding tie lines (dotted lines). The measured liquidus compositions agree reasonably well, as described previously for Figure 5. Table 3 shows the solid solution compositions through the iron-to-nickel ratio, measured via material balance or computed from the thermodynamic model. The average error deviation between experimental and model-calculated solid compositions was 3 wt % for both FeSO_4_ and NiSO_4_, which was determined through inspection of the phase diagram shown in Figure 6.

**Figure 6:**
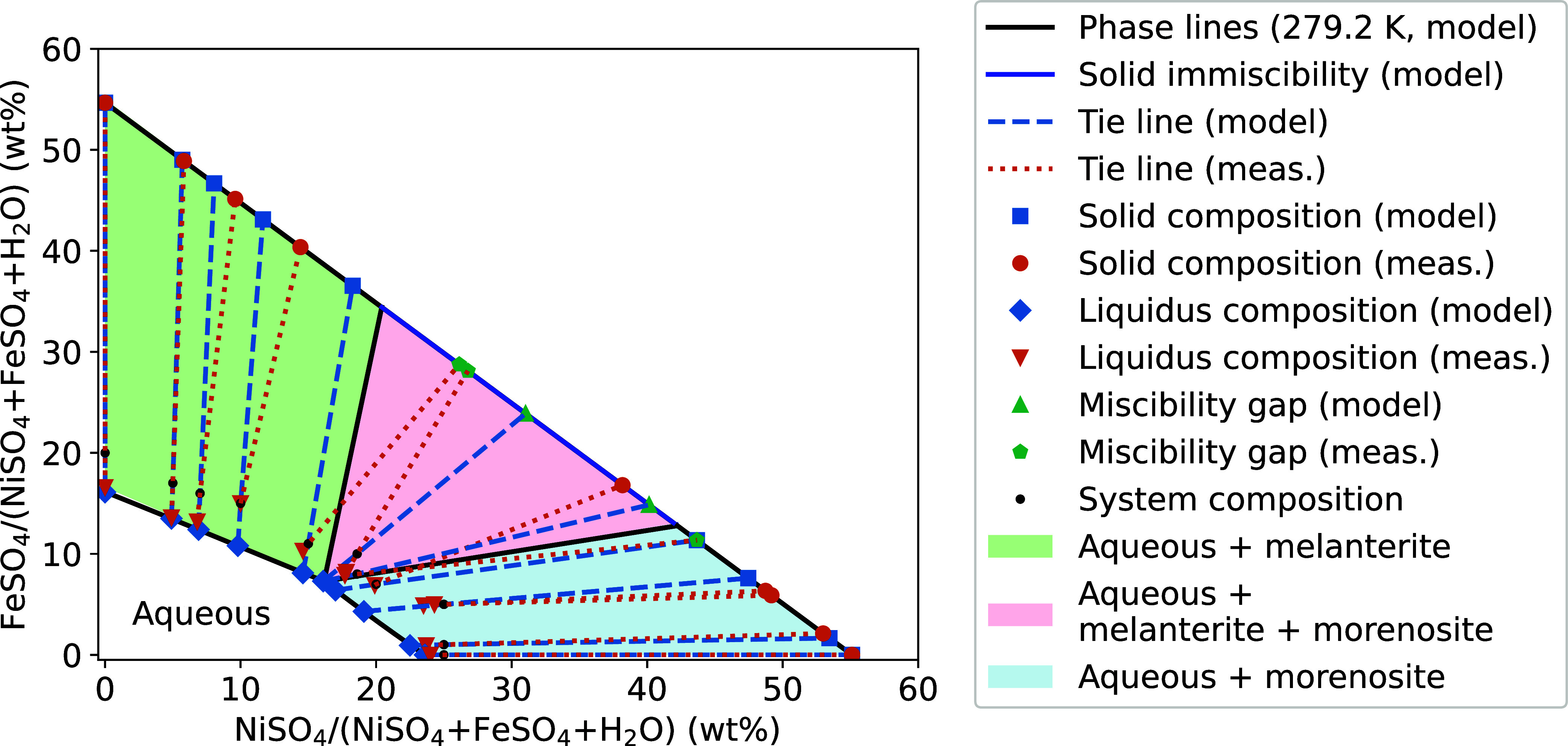
Isothermal ternary phase diagram of FeSO_4_–NiSO_4_–H_2_O. Points computed from the thermodynamic model are shown as squares and diamonds. Points as circles or upside-down triangles are computed from material balance and chemical analysis. Model-calculated data points in the immiscible region (aqueous + melanterite + morenosite, triangles) are not indications of miscibility but of the cumulative composition of all solids present. Numerical values are listed in Tables 2 and 3. The identification of solid immiscibility for measured solid composition points (pentagons) was conducted through XRD. The pressure is atmospheric (100 ± 3 kPa).

**Table 3:** Solid Solution Iron Sulfate to Nickel Sulfate Ratio at Various Temperatures as a Function of Iron and Nickel Sulfate Composition of System ( $\frac{w_{{\mathrm{FeSO}}_{4}}}{w_{\mathrm{Tot}}}$ , $\frac{w_{{\mathrm{NiSO}}_{4}}}{w_{\mathrm{Tot}}}$ ) at Atmospheric Pressure (100 kPa)^a^

*T*	$\frac{w_{{\mathrm{FeSO}}_{4}}}{w_{\mathrm{Tot}}}$	$\frac{w_{{\mathrm{NiSO}}_{4}}}{w_{\mathrm{Tot}}}$	$\frac{\sum_{\mathrm{crystal}}w_{{\mathrm{FeSO}}_{4}}^{\mathrm{crystal}}}{\sum_{\mathrm{crystal}}w_{{\mathrm{NiSO}}_{4}}^{\mathrm{crystal}}}$		Phase(s)	
				
(K)	(wt %)	(wt %)	AAS	model	XRD	model
279.2	20.0	0	–	–	S_1_	S_1_
279.2	17.01	5.00	8	9	N/A	S_1_
279.2	15.99	7.01	4.7	5.8	S_1_	S_1_
279.2	15.00	10.0	2.8	3.7	N/A	S_1_
279.2	11.01	15.0	1.1	2.0	S_1_, S_2_	S_1_
279.2	10.00	18.6	1.1	0.8	N/A	S_1_, S_2_
279.2	8.00	18.6	0.26	0.37	N/A	S_1_, S_2_
279.2	6.99	20.0	0.44	0.26	S_2_	S_2_
279.2	5.00	25.0	0.13	0.16	S_2_	S_2_
279.2	5.00	25.0	0.12	0.16	S_2_	S_2_
279.2	1.01	25.0	0.041	0.031	S_2_	S_2_
279.2	0	25.0	0	0	S_2_	S_2_
281.2	17.00	5.00	8	9	S_1_	S_1_
281.2	15.00	10.0	3.0	3.8	S_1_	S_1_
283.2	10.00	18.6	1.4	1.0	S_1_, S_2_	S_1_, S_2_
281.2	8.00	18.6	0.37	0.39	S_1_, S_2_	S_1_, S_2_

a The solid phases S_1_ (melanterite), S_2_ (morenosite) were identified for each system composition, either from XRD or from the model. Standard uncertainty: *u*(*T*) = 0.6 K, *u*(*P*) = 3 kPa. Relative uncertainties on measured quantities $u_{r}\left(\frac{w_{\mathrm{FeSO}4}}{w_{\mathrm{Tot}}}\right)=0.005$ ; $u_{r}\left(\frac{w_{\mathrm{NiSO}4}}{w_{\mathrm{Tot}}}\right)=0.01$ , $u_{r}(\frac{\underset{\mathrm{crystal}}{\sum }w_{{\mathrm{FeSO}}_{4}}^{\mathrm{crystal}}}{\underset{\mathrm{crystal}}{\sum }w_{{\mathrm{NiSO}}_{4}}^{\mathrm{crystal}}})=0.3$ .

The AAS ratio values allow calculation of the solid solution composition, which can be compared to that computed from the thermodynamic model. Table 3 shows the model-predicted solid phases (melanterite S_1_, morenosite S_2_, melanterite + morenosite S_1_ + S_2_) of the various regions, which are also labeled in Figure 6. Measured solid compositions were, within reported error, in the same regions as the model-calculated solid compositions except for the 11.0 wt % FeSO_4_ and 15.0 wt % NiSO_4_ system, for which the experimental solid composition was in the S_1_ + S_2_ region instead of the S_1_ region. We note that this sample took a week to crystallize, while other samples crystallized under 24 h. Although the solid composition and XRD indicate immiscibility of solids, the liquidus composition is far from the isothermally invariant liquidus point realized by the 10.00 and 8.00 FeSO_4_ wt % at 18.6 NiSO_4_ wt % system compositions. Thus, we speculate that this is due to kinetic limitations in obtaining the thermodynamically stable phases on the time scales of the experiment.

Generally, given the agreement of the experimental solubility limits and the tie lines with those calculated from the thermodynamic model, the revised *K*_SP_ values we suggest for iron in morenosite and nickel in melanterite (see Table 4) and their corresponding thermodynamic properties in Table 5 are adequate to describe the solid–liquid equilibria from 279.2 to 298.2 K. The approximations mentioned above, made to estimate the entropy and enthalpy of a given solid solution, could be corrected in further work by studying the temperature dependence of the solid miscibility gap composition, as well as the heat capacity of the solid solutions. We stress that ideality to model the solid solutions is only an approximation. The nonidealities of the solid solution, which remain to be quantified in further work, would require additional thermodynamic data to be probed and evaluated. The disagreement of the tie lines between experiment and the model reflects the likely errors in the phase diagram and shows that the ideal solution is a reasonable first approximation.

**Table 4:** Solubility Constants for Various Solid Species at 25 °C

	ln(*K*_SP_)	Δ*G* ^0^ (kJ/mol)	ref
FeSO_4_·7H_2_O(s, melanterite basis)	–5.2554	13.0279	^13^
NiSO_4_·7H_2_O(s, morenosite basis)	–5.0222	12.4498	^14^
FeSO_4_·7H_2_O(s, morenosite basis)	–4.2155	10.4500	Present work
NiSO_4_·7H_2_O(s, melanterite basis)	–4.4542	11.0418	Present work
FeSO_4_·7H_2_O(s, morenosite basis)	–2.35	5.8255	^10^
NiSO_4_·7H_2_O(s, melanterite basis)	–3.27	8.1062	^10^

**Table 5:** Thermodynamic Quantities Used in the Model

	Δ*H*_f_^°^(298 K) (J/mol)	*S*°(298 K) (J/mol·K)	ref
FeSO_4_·7H_2_O(s, melanterite basis)	–3017510.0	395.30	^13^
NiSO_4_·7H_2_O(s, morenosite basis)	–2976610.0	379.16	^13^
NiSO_4_·7H_2_O(s, melanterite basis)	–2970389.8	395.30	See text
FeSO_4_·7H_2_O(s, morenosite basis)	–3019744.3	379.16	See text
FeSO_4_·4H_2_O(s, rozenite)	–2131060.0	270.60	^13^
NiSO_4_·6H_2_O(s, α-retgersite)	–2683730.0	331.96	^14^
NiSO_4_·6H_2_O(s, β-hexahydrite)	–2676560.0	353.85	^14^
Fe^2+^(aq)	–92260.0	–105.90	^13^
Ni^2+^(aq)	–53973.6	–128.87	^14^
SO_4_^2–^(aq)	–909340.0	18.50	^13^
H_2_O(l)	–285830	69.95	^13^

**Table 6:** Heat Capacities Used in the Thermodynamic Model

	*C*_*P*_ (J/mol·K)	ref
FeSO_4_·7H_2_O(s, melanterite basis)	379.822 + 0.3619*T* – 8224500/*T* ^2^	^13^
NiSO_4_·7H_2_O(s, melanterite basis)	379.822 + 0.3619*T* – 8224500/*T* ^2^	See text
NiSO_4_·7H_2_O(s, morenosite basis)	106.688 + 0.8655*T*	^14^
FeSO_4_·7H_2_O(s, morenosite basis)	106.688 + 0.8655*T*	See text
Fe^2+^(aq)	14279.4 – 57.8948*T* – 258870000/*T* ^2^ + 0.0659339*T* ^2^	^13^
Ni^2+^(aq)	16343.1 – 66.3614*T* – 296429000/*T* ^2^ + 0.075689*T* ^2^	^14^
SO_4_^2–^(aq)	46200.6 – 186.8004*T* – 854629000/*T* ^2^ + 0.211929*T* ^2^	^13^
H_2_O	–203.119 + 1.5207*T* + 3848757.662/*T* ^2^ – 0.0031913*T* ^2^ + 0.000002471*T* ^3^	FactSage^26^

**Table 7:** Liquid Electrolyte Binary Interaction Parameters for FeSO_4_(aq)

parameter	expression	ref
β^0^	–508.3/*T* + 5.1934 – 0.0161*T* + 1.8349 × 10^–5^ *T* ^2^	^13^
β^1^	–3205.3/*T* + 15.8514 + 0.0085*T* – 6.0442 × 10^–5^ *T* ^2^	^13^
α	1.4	^13^
β^2^	–16.2142	^13^
α	12	^13^
*C* ^ϕ^	12.8/*T* – 0.0588	^13^

In order to verify that the solids in the different phase regions had the crystallographic structures corresponding to those indicated by the model, crystallographic analysis was undertaken. Figure 7 shows diffraction profiles obtained in three different solid formation regions presented above. Given that the XRD sample holder was at room temperature and did not have humidity control, the thermodynamic conditions of the studied solids were not exactly those of the solid–liquid equilibria from which they appeared. As such, they were subject to potential dehydration. The diffractograms showed diffraction patterns implying the presence of melanterite and/or morenosite, as well as rozenite (FeSO_4_·4H_2_O) and nickelhexahydrite (β-NiSO_4_·6H_2_O), but not retgersite (α-NiSO_4_·6H_2_O). We also verified with FactSage software our assumption that these lower hydration phases are not the thermodynamically stable phases that would appear in solid–liquid equilibria at low temperature (e.g., 279.2 to 283.2 K) but are exclusively consequences of the artificial dehydration of melanterite and morenosite in the XRD sample holder. The dehydration of FeSO_4_·7H_2_O has been studied by different authors^4,8,27,28^ who agree that, under conditions with relative humidity (RH) less than 55–65% at room temperature (*T* = 298.2 K), the heptahydrate structure decomposes into the tetrahydrate structure. The thermodynamic properties of rozenite shown in Table 5 are consistent with these dehydration conditions. The dehydration pathway can be described via the following equation:

18 $$FeSO_{4}\cdot 7H_{2}O\left(s\right)\overset{T=298.15\;K}{\underset{\mathrm{RH}\leq 65\%}{\to }}FeSO_{4}\cdot 4H_{2}O\left(s\right)+3H_{2}O\left(l\right)$$

Furthermore, according to Mitchell (1984),^28^ the tetrahydrate structure of rozenite remains stable as long as the above conditions remain unchanged, especially without an increase in temperature to around 313.2 K. A similar logic applies for the dehydration of morenosite. It reportedly only remains stable at ambient temperature at high RH (>85%), and under sufficiently dry conditions, it dehydrates into beta hexahydrate.^29–31^ However, unlike rozenite, nickel hexahydrite does not remain stable, transitioning to retgersite (α-NiSO_4_·6H_2_O) after a certain period of time.^29–31^ Eqs 19 and 20 summarize these dehydration steps:

19 $${\mathrm{NiSO}}_{4}\cdot 7H_{2}O\left(s\right)\overset{T=298.15\;K}{\underset{\mathrm{RH}\leq 85\%}{\to }}{\beta \mathrm{‐NiSO}}_{4}\cdot 6H_{2}O\left(s\right)+H_{2}O\left(l\right)$$

20 $${\beta \mathrm{‐NiSO}}_{4}\cdot 6H_{2}O\left(s\right)\underset{\mathrm{Time}}{\to }{\mathrm{\alpha ‐NiSO}}_{4}\cdot 6H_{2}O\left(s\right)$$

**Figure 7:**
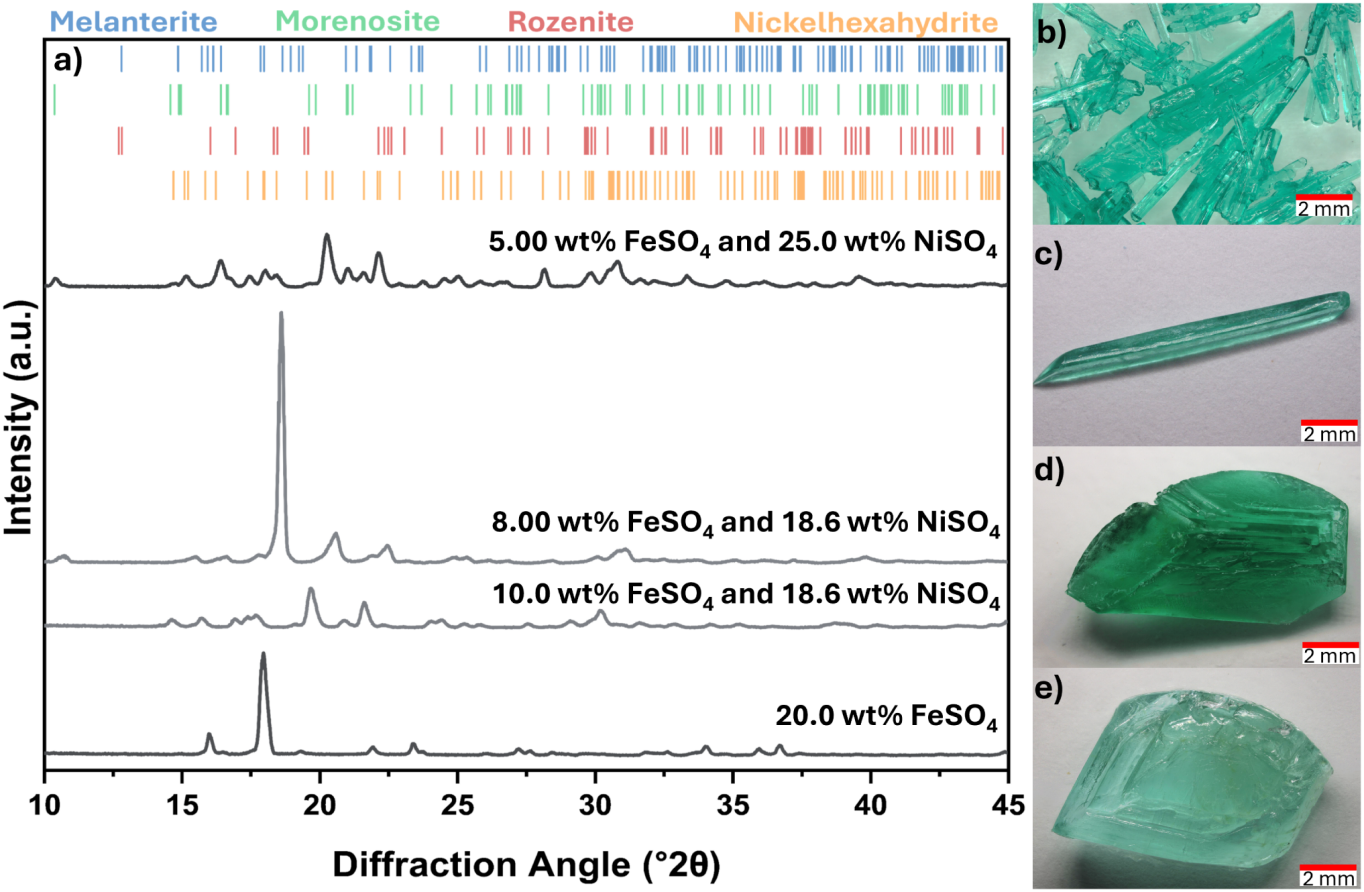
a) XRD diffraction pattern of crystals from different systems. Optical microscope images collected from the experiments: b) 5 wt % FeSO_4_ and 25 wt % NiSO_4_ at 279.2 K, c) 8.00 wt % FeSO_4_ and 18.6 wt % NiSO_4_ at 281.2 K, d) 10.0 wt % FeSO_4_ and 18.6 wt % NiSO_4_ at 283.2 K, and e) 20.0 wt % FeSO_4_ at 279.2 K. The pressure is atmospheric (100 ± 3 kPa). Scale bar: 2 mm. In this work, we assume the nonheptahydrate forms are results of dehydration occurring during the XRD measurements and are not the thermodynamically stable phases (see text).

The thermodynamic properties of nickelhexahydrite and retgersite shown in Table 5 ^13^ are consistent with these dehydration conditions. Although the presence of (Ni, Fe) solid solutions complicates the energetics of eqs 18, 19, and 20, we reasonably assume these still apply, provided the environment is sufficiently dry. As a result of analyzing the crystal structure through Le Bail refinement of each of the crystals formed, the refinement was validated through a χ^2^ value lower than 1.8. Taking into account dehydration, it was possible to identify phase consistency between the model and material balance solid–liquid equilibria. Table 3 shows the phases identified via refinement for the various system compositions (denoted XRD). In the system containing 20.0 wt % FeSO_4_, the melanterite phase was expected. As shown in Figure 7, the diffraction profiles correspond mainly to this phase, with a few minor peaks corresponding to rozenite. As discussed previously, for the two systems containing 18.6 wt % NiSO_4_ with 8.00 or 10.0 wt % FeSO_4_, the presence of melanterite and morenosite was expected, and the diffractograms indicate the presence of both phases. For certain system compositions including these, XRD was carried out only at temperatures of 281.2 and 283.2 K (see Table 3). Although an approximation, we assume that temperature variations between 279.2 and 283.2 K do not significantly affect the solid phases identified. The signal of the system containing 10.0 wt % FeSO_4_ showed some morenosite dehydration, while the system with 8.00 wt % FeSO_4_ did not indicate either of the two phases (rozenite or nickelhexahydrite). Finally, Figure 7 presents the system with 5.00 wt % FeSO_4_ and 25.0 wt % NiSO_4_ which appears in Figure 6 in the region where only the S_2_ phase is stable. As expected, only peaks associated with morenosite and the phase resulting from its dehydration, nickel hexahydrite, were identified.

**Table 8:** Liquid Electrolyte Binary Interaction Parameters for NiSO_4_(aq)

parameter	expression	ref
β^0^	–75.73582/*T* + 0.40892	^14^
β^1^	–1192.39972/*T* + 7.02089	^14^
α	1.4	^14^
β^2^	–79106.27192/*T* + 595.45536 −1.23307*T*	^14^
α	12	^14^
*C* ^ϕ^	41.08029/*T* – 0.09686	^14^

Figure 7 also presents the morphologies corresponding to the diffraction patterns shown. Despite the presence of rozenite and nickelhexahydrite in its patterns, it is expected that the morphology of the crystals will be mainly associated with melanterite, morenosite, or a combination of these two phases, since dehydration occurring in the present work is not representative of the solid–liquid equilibria under study. Melanterite can be described as a blue-green translucent crystal with a quadrangular prismatic shape.^32,33^ Morenosite, on the other hand, is described as a green needlelike, elongated, or short prismatic crystal.^3,34^ Although the focus of this study was not to determine the crystal morphology by considering nucleation and kinetics, it was possible to observe a correlation between the literature description and the crystals obtained experimentally in this work in regions where the formation of a single phase was expected. In this context, Figure 7a and e shows optical microscope images of the crystals formed in these regions, representing, respectively, the 20.0 wt % iron sulfate system in the melanterite formation region and the 5.00 wt % iron sulfate and 25.0 wt % nickel sulfate system in the morenosite formation region. The two systems containing 18.6 wt % nickel sulfate exhibit morphologies intermediate between the melanterite and morenosite phases. The solid composition of the system containing 10.0 wt % iron sulfate in Figure 6 is richer in melanterite; accordingly, it exhibits a morphology closer to that observed for the crystal containing only this phase (20.0 wt % iron sulfate) in Figure 7c. In Figure 7d, the system with 8.00 wt % iron sulfate, whose solid composition is richer in morenosite, has a morphology similar to that presented for the crystal containing only this phase (5.00 wt % iron sulfate and 25.0 wt % nickel sulfate).

Figures 8 and 9 show the remaining diffraction profiles and their respective morphologies, which also showed peak signals associated with crystal dehydration. In accordance with the model-calculated phase diagram shown in Figure 6, the expected phases were obtained from the diffractograms, except for the 11.0 wt % FeSO_4_ and 15.0 wt % NiSO_4_ system. While the model predicted that the solid should be strictly melanterite, both melanterite and morenosite were experimentally observed. The morphologies are accordingly those expected from the XRD-inferred phases. In Figure 8e and d, the systems with 17.0 and 15.0 wt % FeSO_4_ showed quadrangular prismatic crystal shapes, and for Figure 8c 11.0 wt % FeSO_4_ system, the sample also has a prismatic shape but with elongated edges, consistent with the description of morenosite.^3^

**Table 9:** Liquid Electrolyte Ternary Interaction Parameters for FeSO_4_(aq) + NiSO_4_(aq)

parameter	value	ref
$\Psi_{Ni^{2+},Fe^{2+},{{\mathrm{SO}}_{4}}^{2-}}$	0.05	^10^

**Figure 8:**
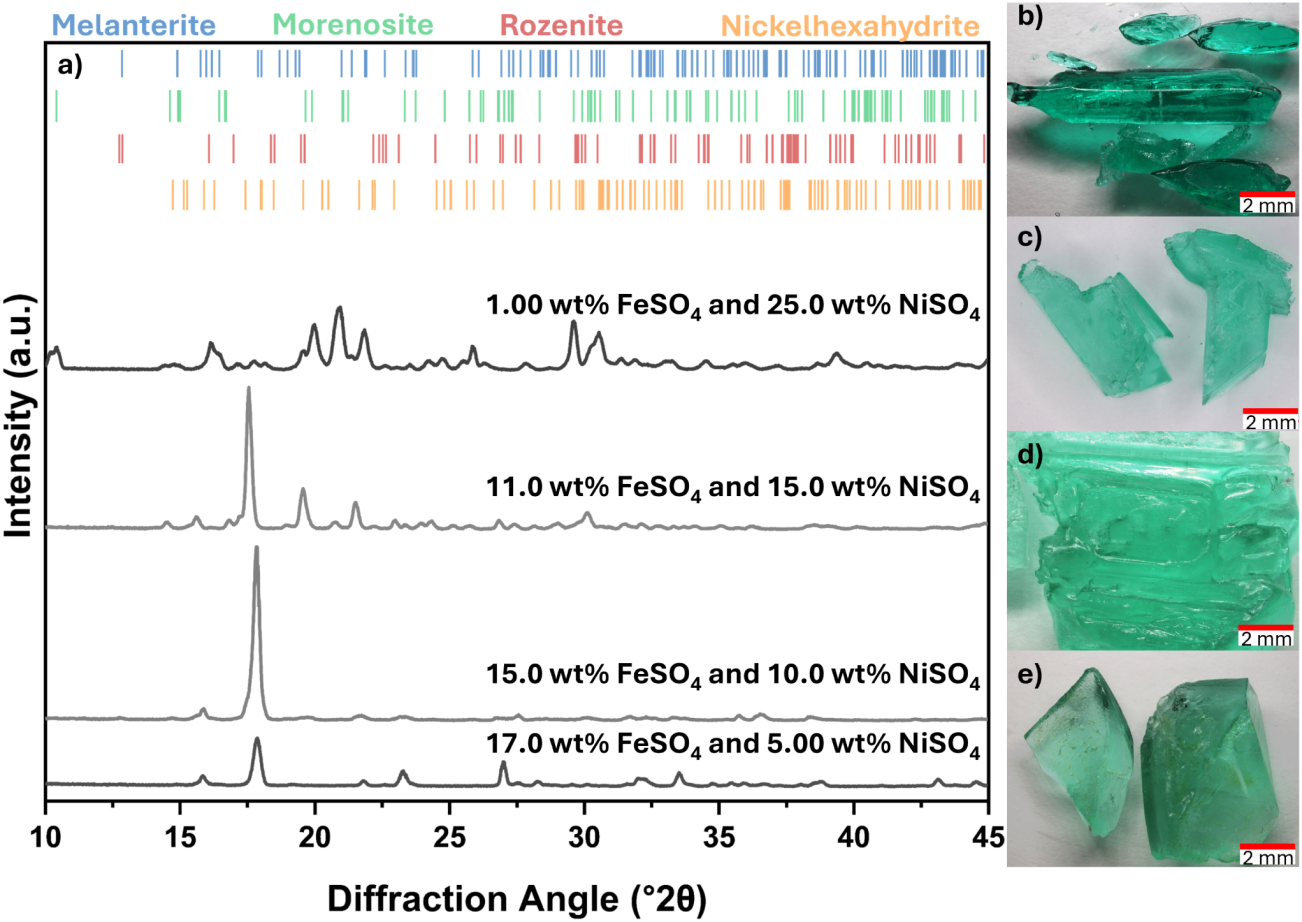
a) XRD diffraction pattern of crystals from different systems. Optical microscope images collected from the experiments: b) 1.00 wt % FeSO_4_ and 25.0 wt % NiSO_4_ at 279.2 K, c) 11.0 wt % FeSO_4_ and 15.0 wt % NiSO_4_ at 279.2 K, d) 15.0 wt % FeSO_4_ and 10.0 wt % NiSO_4_ at 281.2 K, and e) 17.0 wt % FeSO_4_ and 5.00 wt % NiSO_4_ at 281.2 K. The pressure is atmospheric (100 ± 3 kPa). Scale bar: 2 mm. In this work, we assume the nonheptahydrate forms are results of dehydration occurring during the XRD measurements and are not the thermodynamically stable phases (see text).

**Figure 9:**
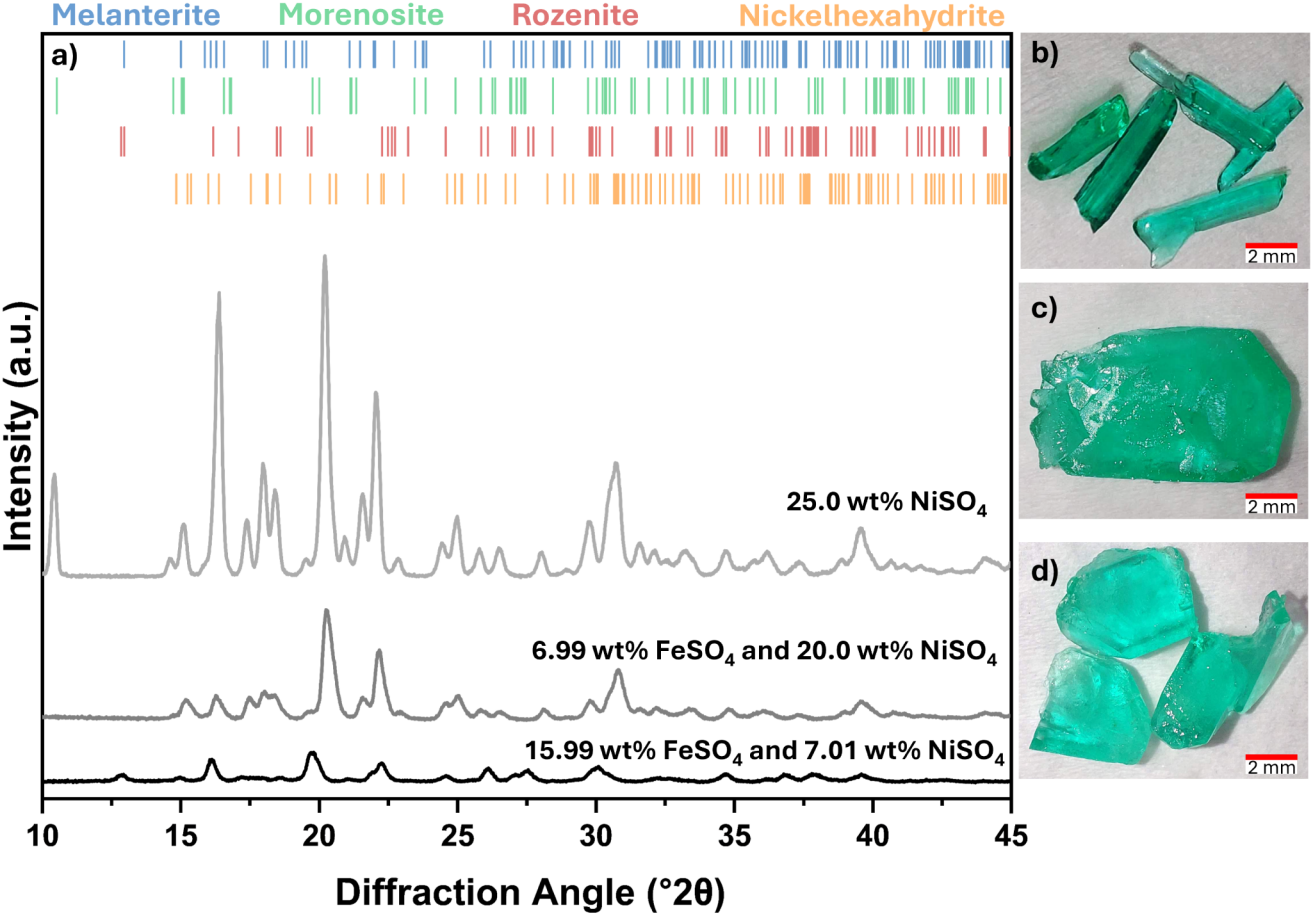
a) XRD diffraction pattern of crystals from different systems. Optical microscope images collected from the experiments: b) 25.0 wt % NiSO_4_ at 279.2 K, c) 6.99 wt % FeSO_4_ and 20.0 wt % NiSO_4_ at 279.2 K, and d) 15.99 wt % FeSO_4_ and 7.01 wt % NiSO_4_ at 279.2 K. The pressure is atmospheric (100 ± 3 kPa). Scale bar: 2 mm. In this work, we assume the nonheptahydrate forms are results of dehydration occurring during the XRD measurements and are not the thermodynamically stable phases (see text).

## Conclusions

From this work, it was possible to assess the aqueous system of iron and nickel sulfates in terms of solid–liquid equilibria as well as the presence of solid solutions. The methodology used, which considers cooling the solutions to temperatures below the modeled solid–liquid transition, allowed us to calculate the liquidus composition and solid phases based on the chemical characterization of the crystal through material balance. The collected data were consistent with those computed from the thermodynamic model, where the average disagreement regarding the liquidus composition was on the order of 1 wt % and regarding the solid composition 3 wt % for each of the sulfates. Thus, it was possible to conclude that the experiments and model fairly described the solid–liquid equilibria. The assumption that the system would solidify as a solid solution was verified through the crystal composition and found to be consistent with the XRD measurement. Improving our experimental setup to include an XRD sample holder with humidity control would allow better resolution of crystalline phases and the miscibility gap. Two significant modeling improvements would be possible through vapor pressure measurements of saturated aqueous solutions at different temperatures. This would allow fitting temperature-dependent Pitzer parameters in addition to allowing access to solid-solution energetics and nonidealities through measured tie lines.

Since data are lacking on the possible solid solution formation of hydrates that are relevant at higher temperatures, we believe their study would be fruitful in the effort to use solid–liquid equilibrium in controlled chemical precipitation and will be the subject of future work.
